# Anticancer Peptide Prediction *via* Multi-Kernel CNN and Attention Model

**DOI:** 10.3389/fgene.2022.887894

**Published:** 2022-04-27

**Authors:** Xiujin Wu, Wenhua Zeng, Fan Lin, Peng Xu, Xinzhu Li

**Affiliations:** ^1^ School of Informatics, Xiamen University, Xiamen, China; ^2^ Boston Children’s Hospital, Boston, MA, United States; ^3^ Chongqing Michong Technology Co., Ltd., Chongqing, China

**Keywords:** anticancer peptide, multi-CNN, attention mechanism, prediction, classfication

## Abstract

**Background:** Modern lifestyles mean that people are more likely to suffer from some form of cancer. As anticancer peptides can effectively kill cancer cells and play an important role in fighting cancer, they have been a subject of increasing research interest.

**Methods:** This study presents a useful tool to identify the anticancer peptides based on a multi-kernel CNN and attention model, called ACP-MCAM. This model can automatically learn adaptive embedding and the context sequence features of ACP. In addition, to obtain better interpretability and integrity, we visualized the model.

**Results:** Benchmarking comparison shows that ACP-MCAM significantly outperforms several state-of-the-art models. Different encoding schemes have different impacts on the performance of the model. We also studied tmethod parameter optimization.

**Conclusion:** The ACP-MCAM can integrate multi-kernel CNN and self-attention mechanism, which outperforms the previous model in identifying anticancer peptides. It is expected that the work will provide new research ideas for anticancer peptide prediction in the future. In addition, this work will promote the development of the interdisciplinary field of artificial intelligence and biomedicine.

## Introduction

Anticancer peptide (Plumb et al., 2019) (ACP) is a polypeptide sequence with anticancer activity. It is composed of 10–50 amino acid amino acids (Barras and Widmann, 2011). Its molecular structure is complex. It is a molecular polymer between amino acids and proteins (Li et al., 2006), which is composed of several or dozen amino acids connected by peptide bonds (Gaspar et al., 2013). It can destroy the structure of the tumor cell membrane and inhibit the proliferation and migration of cancer cells (Song et al., 2020). It can induce the apoptosis of cancer cells without damage to normal human cells (Qiao et al., 2019; Ryu et al., 2021). At present, most anti-cancer drugs have side effects on the kidney (Kamisli et al., 2015; Van Acker et al., 2016), nerve and heart (Plumb et al., 2019), and gonads (Novin and Sciences, 2014; Gutierrez et al., 2016). Compared with conventional chemotherapy, anticancer peptides have the advantages of high specificity, low production cost, high tumor penetration rate, and easy synthesis and modification (Otvos, 2008). Due to the benefits of anti-cancer peptides, more and more anti-cancer peptides are used in clinical trials. For example, three peptides Didemin A, B, and C, which are extracted from sea squirt, have obvious inhibitory effects on breast cancer, ovarian cancer, and uterine cancer, and have entered phase II clinical trials (Clamp and Jayson, 2002). The synthetic peptide Elisidepsin (PM02734) has also entered phase II clinical trials (Ratain et al., 2015). Identifying anti-cancer peptides is of great significance for discovering new and efficient treatments for diseases. Traditional anti-cancer peptide prediction relies on biological experiments, and the prediction is accurate, but it is inefficient, time-consuming, and costly. With the development of Qualcomm sequencing technology, protein sequence data is increasing exponentially every year, and massive sequence data presents severe challenges to biological experiment technology (Ratain et al., 2015).

Some research methods have used machine learning and deep learning methods to build anticancer peptide prediction models (Su et al., 2019; Su et al., 2020; Wei et al., 2020). Tyagi et al. proposed an anti-cancer peptide prediction method called AntiCP (Tyagi et al., 2013), which uses amino acid composition, dipeptide composition, and composition differences between amino acid N-terminus and C-terminus, combined with a support vector machine (SVM) model for prediction. Chen et al. (Chen et al., 2016) used the features of amino acid dipeptide composition and pseudo-amino acid composition, combined with a support vector machine to construct an anti-cancer peptide prediction algorithm called iACP (Chen et al., 2016). Wei et al. have proposed the ACPred-FL (Leyi et al., 2018) model and the PEPred-Suite model (Leyi et al., 2019). The ACPred-FL (Leyi et al., 2018) model used four sequence feature representation samples: binary profile features (BPF), G-gap dipeptide composition (GDC), overlapping property (OPF), and composition transition distribution (CTD), and the frequency of each amino acid in the sequence, combined with the SVM model to build 40 sub-models, and then the output of the 40 sub-models are used as the input feature to build the model for anti-cancer peptide prediction. AntiCP 2.0 (Agrawal et al., 2020) uses SVM, ETree, random forest, ridge algorithm, artificial neural network (ANN), and the K nearest neighbor (KNN) method to construct the prediction model of anticancer peptides. There have also been integrated anti-cancer peptide prediction methods, which integrate multiple or multiple machine learning methods to predict various peptide sequences (Li et al., 2017; Wei et al., 2017; Leyi et al., 2018).

Existing models have some problems, such as low recognition accuracy, insufficient generalization ability, and there is a lack of large-scale evaluation of features and prediction models. Almost all the existing anticancer peptide prediction studies use sequence features to construct anticancer peptide prediction models, which show that the anticancer peptide prediction methods based on sequence information are effective. But most research to date has not considered the combination of protein structure and sequence data characteristics or the feature space used was not comprehensive enough. Most of them use single machine learning methods, such as support vector machines, and seldom use the attention mechanism (Vaswani et al., 2017). To solve the above problems, this article takes the anti-cancer peptide sequence data as the research object, exploring the anti-cancer peptide prediction method based on the attention mechanism (Vaswani et al., 2017) and deep learning models, establishing a relatively advanced and effective anti-cancer peptide prediction model. The main research contents are summarized as follows:

1) The paper proposes a model with strong anti-cancer peptide recognition ability based on learnable adaptive embedding and amino acid structure features. It is a self-attention mechanism that can automatically learn the context sequence features of ACP, learns the contribution of each amino acid node in the entire anti-cancer peptide sequence, automatically captures the global information in the ACP sequence, and can capture the contributions of protein cluster formed by 3–5 amino acid nodes in the anti-cancer peptide sequence to improve the ability to identify the anti-cancer peptide model.
2) This article comprehensively evaluates the different feature projects of anti-cancer peptides, constructs a deep learning model with strong performance, and integrates the advantages of multiple deep learning models to improve predictive performance. To improve the interpretability of the model prediction results, this article visualizes model prediction characteristics to improve the interpretability of the model prediction results.
3) The methods for identifying various functional peptides of the same type from the same functional peptides are relatively similar. It is difficult to identify anti-cancer peptides from multiple peptide sequences. Most of the existing methods recognize anti-cancer peptides from a single type of peptide sequence, so research on new deep learning models is needed. The new deep learning model studies the impact of different coding schemes on the performance of the model, examining the method of parameter optimization to build a better deep learning model and obtain the best model for identifying anticancer peptides from different functional peptides.

This article first examines the peptide datasets used, which are introduced in *Datasets Section*. Second, the anticancer peptide predicting model of multi-kernel CNN and the attention mechanism is explained in detail in *Model Overview Section*. Third, the performance evaluation index, loss function, experimental process, and results of the model are presented in *Experiments and Results Section*. Finally, the results and the prospects for future work are discussed and summarized in *Conclusion Section*.

## Materials and Methods

This chapter introduces the structural frame of the ACP-MCAM model and the datasets of anti-cancer peptides in detail.

### Datasets

The datasets used in this experiment come from the literature (Leyi et al., 2018). The samples in this dataset were also collected from literature (Tyagi et al., 2013; Atul et al., 2015). Among them, the positive samples are anti-cancer peptides that have been confirmed by physical experiments, and the negative samples are selected from anti-microbial peptides that have no anti-cancer activity. The training dataset ACPs500 consists of 250 anti-cancer peptides and 250 non-anti-cancer peptides. The test dataset ACPs164 consists of 82 positive samples and 82 non-anticancer peptide samples. All of the samples are filtered by CD-HIT (Ying et al., 2010) to filter out redundant sequences with a similarity higher than 90% so that the sequences in the training dataset and the test dataset are different. Two other data sets, neuropeptides and antifungal peptides, were also used in this paper. The details of the dataset are shown in Table 1.

**TABLE 1:** Summary of datasets.

Datasets	Dataset Type	Total Number	Number of Positive Samples	Number of Negative Samples
ACPs500	Training set	500	250	250
ACPs164	Test set	164	82	82
NPs1400	Training set	1400	700	700
NPs350	Test set	350	175	175
AFPs2336	Training set	2336	1168	1168
AFPs582	Test set	582	291	291

### Model Overview

This chapter details the ACP-MCAM model structural frame used to predict the anticancer peptides.

**FIGURE 1:**
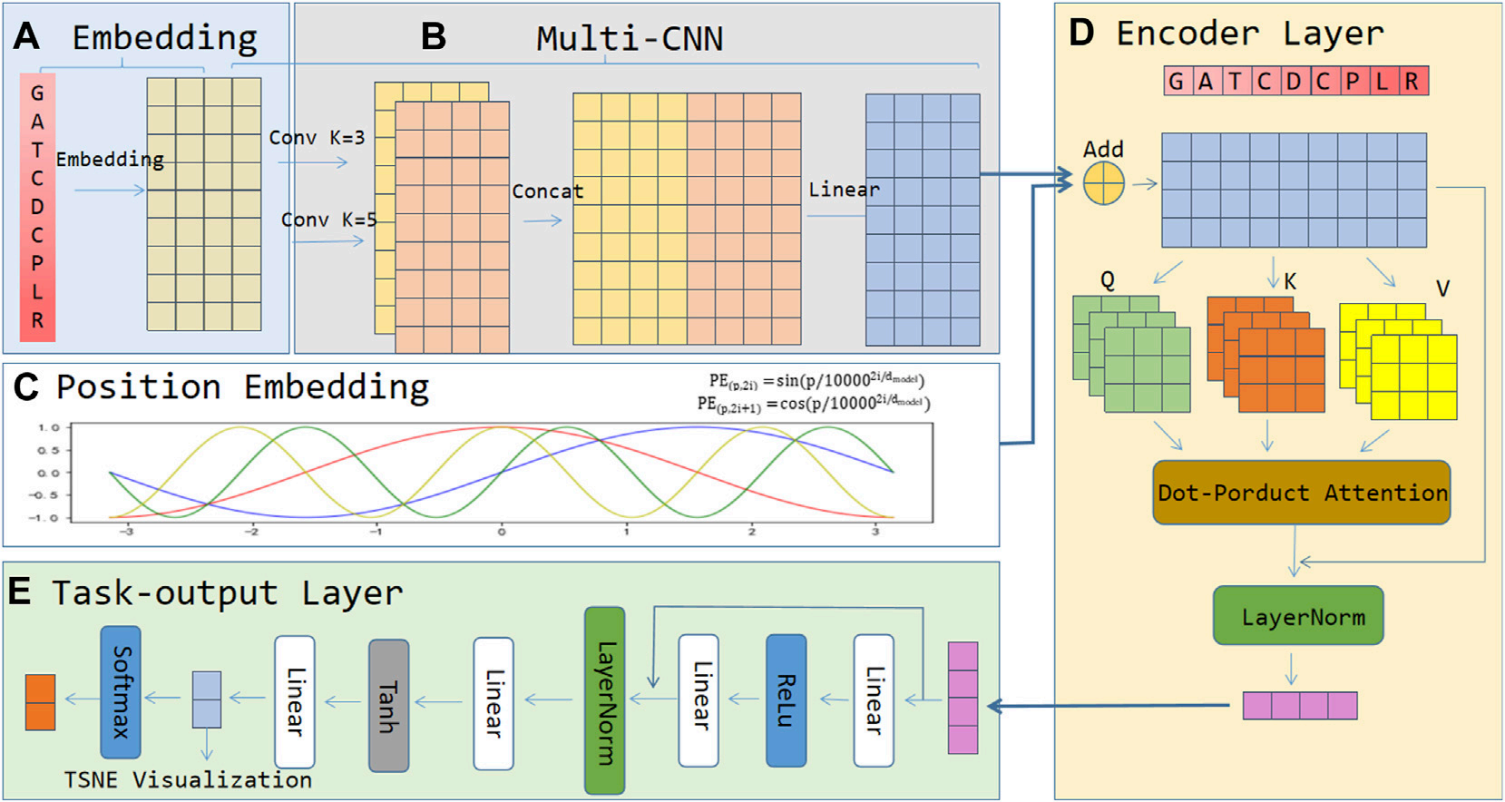
The framework of the proposed ACP-MCAM. **(A)** Embedding layer. **(B)** Multi-kernel CNN layer. **(C)** Position embedding layer. **(D)** Encoding layer. **(E)** Task-output layer.

The architecture of the ACP-MCAM model is shown in Figure 1. It consists of five modules, 1) an embedding layer, 2) a Multi-kernel CNN layer, 3) the position embedding layer, 4) the encoder layer, and 5) the task output layer. In module 1), the embedding layer first processes the input anticancer peptide sequence and converts each amino acid of the anticancer peptide sequence into a low dimensional dense vector as the embedding vector representation of amino acid nodes. No matter where the amino acid appears in the sequence, the same type of amino acid uniquely corresponds to the same vector. 2) In the multi-kernel CNN layer, this paper uses convolution neural network (CNN) technology to encode the amino acid nodes of anticancer peptide sequence by using the context information and different semantic information of specific amino acids in the anticancer peptide sequence. We perform a two-dimensional convolution operation with padding on the output of the embedding layer to ensure that the dimensions of the input and output are the same. The kernel can take odd numbers such as 1, 3, and 5, connect them in the last dimension, and then do a linear transformation. 3) The position embedding layer encodes the position information of the amino acids in the anti-cancer peptide sequence. It is a vector containing the position embedding information of the amino acid sequence. 4) The encoding layer is the core of the model, and the input feature matrix is the output of the position embedding layer and the multi-kernel CNN layer. The encoder layer includes multiple encoder blocks. Each encoder block is based on a multi-head attention mechanism and a fully connected neural network. The feed forward part of each encoder block ensures that the input and output sizes of each encoder block are consistent. The sequential stacking of multiple coding layers makes the representation of anticancer peptide sequences more effective. In module 4), the encoder layer is used to capture the context of each remaining embedding vector at different positions, so that the remaining embedding has different feature vectors according to the context, and learning the discriminative features of ACP. Finally, module 5) is the last part of the model, called the task output layer, which is composed of a fully connected neural network and a nonlinear activation function. It converts the representation of ACPs into the probability distribution of the classes for prediction. Please note that the penultimate neural network in the task output layer is specifically designed for feature visualization. These five modules are described in detail below.

#### Embedding Layer

The core idea of embedding is to map all of the amino acids in the anticancer peptide sequence into a dense vector in a low-dimensional space (mostly *K* = 50–300 dimensions). Since the embedding layer maps each amino acid into a K-dimensional vector. If there are n amino acids in all of the anti-cancer peptide sequences. All of the peptide sequences can be represented by an N×K dimensional matrix. In this article, the anticancer peptide sequences are consist of 20 different amino acids ('A','C','D','E','F','G','H','I','K','L','M','N','P','Q','R','S','T','V','W','Y'). It is not enough to use the underlying embedding feature as the representative feature of the original peptide sequence, a higher level feature needs to be processed on this basis.

#### Single CNN

Modern convolutional neural networks were proposed by LeCun (Lecun et al., 1998). They show excellent performance in solving computer vision problems such as image classification, recognition, and understanding (Farabet et al., 2013; Wang et al., 2017; Yu et al., 2017). The chief conception of the convolutional neural network (CNN) is to capture the local features of the object. At first, it achieved great success in the image field, and later it has also been widely used in the text field. For the anti-cancer peptide data, the local feature is the sliding window composed of several amino acids which is similar to N-gram. The advantage of the convolutional neural network is that it can automatically combine and filter features to obtain semantic information at a different level. For an anticancer peptide sequence “GATCDCPLR”, if kernel = n, the features of n consecutive amino acids on the anticancer peptide sequence can be extracted by convoluting the amino acid of the anticancer peptide sequence. Different kernels can gain different combinations of semantic information of the amino acid in the anticancer peptide sequences. Since each step of the convolution uses the weight sharing mechanism, the training speed is relatively fast. In this article, we use the weight sharing mechanism of CNN to extract the feature of anticancer peptides which have achieved good effects.

#### Multi-Kernel CNN Layer

**FIGURE 2:**
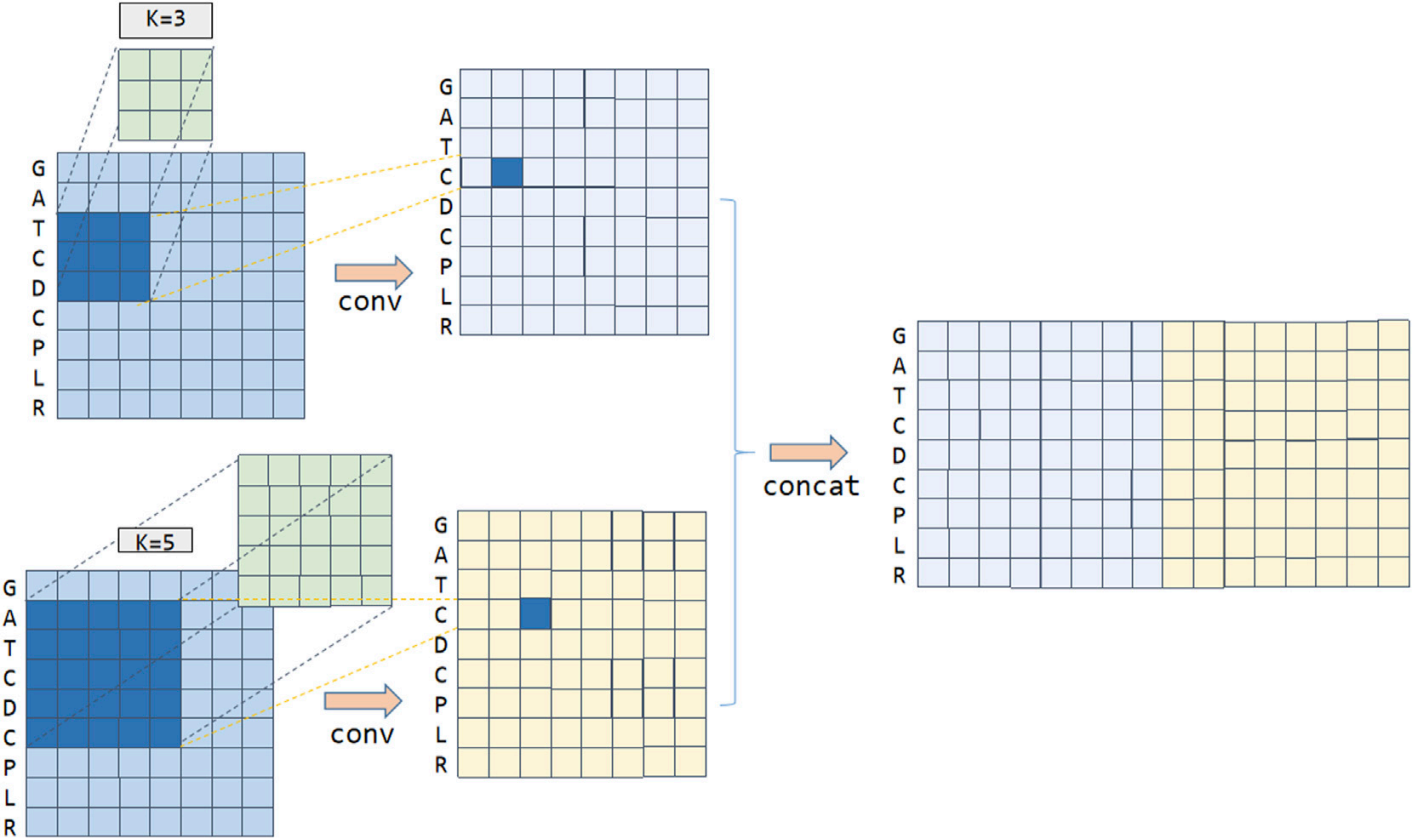
Multi-kernel CNN to extract anticancer peptide sequence features.

The Multi-kernel CNN layer mainly connects multiple convolution kernels of different lengths and combines different anticancer peptide amino acids to obtain different semantic information. For example, for an anti-cancer peptide sequence “GATCDCPLR”, if kernel = 3, the amino acid letter “C” on the anti-cancer peptide sequence is convoluted, and the result of convolution features with sky blue color can be obtained, which is shown in Figure 2. If kernel = 5, the amino acid letter “C” on the anti-cancer peptide sequence is convoluted, and the result of convolution features with yellow color can be obtained, as shown in Figure 2. We concatenate these two convolution results to obtain the convolution features in the first dimension. Convolution kernels of different lengths act on the output matrix of the anti-cancer peptide sequence embedding layer, which can capture different semantic length information and combine them as the input of the deep network.

#### Position Embedding Layer

The original input of the model is an embedding vector without the position information of the amino acid, and the position encoding layer combines the position information with the amino acid embedding vector to form new features and then input into the model. If an anti-cancer peptide sequence of length n is input, the encoding mode of formula (1) (2) can output a unique position code for each time step. Moreover, the distance between any two time steps between anticancer peptide sequences with different lengths is the same.

$$PE_{\left(p,2i\right)}=\sin\left(p/10000^{2i/d_{\mathrm{mod}el}}\right)$$ (1)

$$PE_{\left(p,2i+1\right)}=\cos\left(p/10000^{2i/d_{\mathrm{mod}el}}\right)$$ (2)

Among them, position embedding (PE) indicates the code containing the specific position information of the anticancer peptide after being coded. P represents the position of the amino acid in the sequence, $d_{\mathrm{mod}el}$ represents the dimension of the position vector, and $i\in \left[0,d_{\mathrm{mod}el}\right]$ represents the *i*th dimension of the position $d_{\mathrm{mod}el}$ dimensional position vector. So according to the above formula, we can get the position embedding vector of the *p*th amino acid.

#### Encoding Layer

The basic module of the encoder layer is the encoder model of the transformer (Vaswani et al., 2017). Each encode block includes a multi-head attention mechanism, a feed forward network, and two residual connections. Multi-head attention consists of several self-attention mechanisms, which are used to learn the contextual representation of the sequence. If there are 3 heads, then we linearly transform the features of the anticancer peptide sequence to get the query vector (q1, q2, q3), key vector (k1, k2, k3), and value vector (v1, v2, v3). The query and key calculate the correlation score, named attention score, and then the value is weighted and summed according to the attention score as shown in formula (3) (4).

$$\{\begin{array}{c} Q=XW^{Q}\\ K=XW^{K}\\ V=XW^{V} \end{array}$$ (3)

$$Attention\left(Q,K,V\right)=soft\max\left(\frac{QK^{T}}{\sqrt{d_{k}}}\right)V$$ (4)

Suppose we now want to know the attention score of the first amino acid node “G" in the anticancer peptide sequence. We calculate the respective attention score by the embedding vector of the amino acid node “G" and the embedding vectors of all of the other amino acid nodes in the anticancer peptide sequence. These scores determine the attention weight of the amino acid “G" node embedding vector when we encode the node “G".

In the first step, the query, key, and value vectors are obtained by multiplying the anticancer peptide embedding vector with three parameter matrices. These three parameter matrices are the parameters that the model needs to learn. The second step is to multiply Q by the transpose of K to obtain an initial attention score. Then divide each score by $\sqrt{d_{k}}$ , $d_{k}$ is the dimension of the key vector which is used to make the model more stable when calculating the gradient when backpropagating. The third step is to pass these scores through a softmax function. The softmax function can normalize the scores into probability representation.

#### Task-Output Layer

The output vector of the encoder layer is the feature of anticancer peptides. The main job of the task-output layer is to convert the output vector to binary classification. The task-output layer mainly contains several important modules: linear connection layer, residual network layer, normalization layer, and activation function.

The linear connection layer is a fully connected neural network. It obtains the output of the specified dimension through the linear change of the previous step and plays the role of transforming the dimension. The final dimension corresponds to the number of output categories.

The residual network layer is implemented in the form of skip layer connections, and the input unit is directly added to the output unit. The residual network can solve the degradation problem of the deep neural network well. The residual network converges faster than the same number of layers.

The normalization layer is a standard network layer required by the deep network model. As the number of network layers increases, the output value will become too large or too small. It may cause abnormal and the model may converge very slowly. The normalization layer is used to normalize the output value. Then the output value can be in a reasonable range.

The main role of the activation function is to provide the nonlinear modeling capability of the network. To avoid the pure linear combination, we add an activation function (tanh, ReLU, Softmax, etc.) after the output of each layer. ReLU can keep the gradient undecayed when x > 0, thereby alleviating the problem of gradient disappearance. The output mean of tanh is 0, and its convergence speed is fast, which can reduce the number of iterations. Using different combinations of activation functions can make the network achieve better results.

#### Focal Loss Function

Focal loss (FL) is mainly a strategy proposed by Kaiming to solve the classification problem of indistinguishable samples, that is, to set weights according to the contribution of difficult and easy samples to the loss. The formula is as follows:

$$FL\left(p_{t}\right)=-\alpha_{t}{\left(1-p_{t}\right)}^{\gamma }\log\left(p_{t}\right)$$ (5)

where, $\alpha_{t}$ and γ are parameters that are used to coordinate control samples that are difficult to distinguish, and $p_{t}\;$ represents the probability of ground-truth class. The easier the sample is to distinguish, the larger $p_{t}$ is, and the smaller contribution to the loss. Conversely, the greater the loss of the hard-to-separate sample. This article uses the parameter $\alpha_{t}=0.3\;and\text{ γ}=2\;$ setting to achieve the best results.

## Experiments and Results

In this section, the performance of the ACP-MCAM model is evaluated in several evaluation metrics. We compare it with other models and discuss the results. The experimental parameters are then discussed to verify the effectiveness of the model.

### Evaluating Metrics

The following evaluation indicators were used to evaluate our model. Including recall, precision, accuracy (ACC), Matthew correlation coefficient (MCC), and the area under the ROC curve (AUC) (Balachandran et al., 2018; Leyi et al., 2020; Mehedi et al., 2020). The specific formula is as follows:

{Sensitive=Recall=TPTP+FN=TPP×100%Specificity=TNTN+FP=TNN×100%Precision=TPTP+FP×100%Accuracy= TP+TNTP+TN+FP+FN=TP+TNP+N×100% MCC=TP×TN−FP×FN(TP+FN)(TP+FP)(TN+FN)(TN+FP)×100%       F1=2×precision×recallprecision+recallTPR=TPTP+FN×100%FPR=FPFP+FP×100%Correct index=(TPR+1−FPR)/2 (6)

Among them, true positive (TP) and false negative (FN) represent the number of true anticancer peptides that are predicted correctly and incorrectly. True negative (TN) and false positive (FP) represent the number of non-anticancer peptides that are predicted correctly and incorrectly. Accuracy is the percentage of correctly classified samples in all samples. The sensitivity (SE) and specificity (SP) index measure the predictive ability of predictors for positive and negative samples, respectively. Precision represents the prediction success rate of positive samples. The recall represents the proportion of predicting positive samples in all true positive samples. The F1 score is the coordinated average value of accuracy and recall. The higher the selected value, the better the performance of the model. The other two indicators, AUC and MCC measure the overall performance of the predictor. AUC sorts all samples obtained from model evaluation by score. By calculating the area enclosed by the ROC curve, the AUC value can be obtained.

### Comparison Between ACP-MCAM and Existing Models in Ten-fold Cross Validation

To verify the predictive performance of anticancer peptide ACP-MCAM, we compared it with several existing models, including iACP(Chen et al., 2016), ACPred-FL (Leyi et al., 2018), PEPred-Suite (Leyi et al., 2019), ACPred-Fuse (Rao et al., 2020), AntiCP_ACC (Vijayakumar and Ptv, 2015), AntiCP_DC(Vijayakumar and Ptv, 2015) and Hajisharifi’s(Hajisharifi et al., 2014). The cross validation results are shown in Table 2.

**TABLE 2:** Cross validation results of ACP-MCAM and existing models.

Methods	SE (%)	SP (%)	Accuracy (%)	MCC (%)	AUC (%)
iACP	57.2	84.0	70.6	42.8	80.9
ACPred-FL	71.6	84.4	78.0	56.5	84.6
PEPred-Suite	72.8	88.0	80.4	61.5	86.0
ACPred-Fuse	77.2	87.6	82.4	65.2	88.2
AntiCP_ACC	66.8	78.4	72.6	45.5	82.4
AntiCP_DC	71.6	77.6	74.6	49.3	82.5
Hajisharifi’s	67.2	83.6	75.4	51.5	83.1
ACP-MCAM	**85.6**	**95.2**	**90.4**	**81.3**	**91.9**

Note: The best results are marked in bold and the second best results are underlined.

As shown in Table 2, we can see that the performance of our proposed method ACP-MCAM on all indicators (SE, SP, Accuracy, MCC, and AUC) is significantly better than other predictors, reaching 85.6, 95.2, 90.4, 81.3, and 91.9%, respectively. SE, SP, Accuracy, and MCC are 8.4, 7.8, 8, and 16.1%, which is higher than other predictors.

### Comparison Between ACP-MCAM and Existing Models in Independent Test

In order to verify the superiority of the proposed ACP-MCAM model, we used an independent test dataset to compare its performance with several existing predictions. As shown in Table 3, we can see that the performance of our proposed method ACP-MCAM on all indicators is significantly better than other predictors. SE、SP、ACC、MCC和AUC have reached 85.4, 96.3, 90.9, 82.2 and 94.8%, respectively. Especially SE, MCC, and AUC are 13.4, 50.2, and 8% higher than other predictors. In general, independent test results confirm that our prediction method performs better than other prediction methods, and can better distinguish true anti-cancer peptides from non-anti-cancer peptides.

**TABLE 3:** Independent test results of ACP-MCAM and existing models.

Methods	SE (%)	SP (%)	Accuracy (%)	MCC (%)	AUC (%)
iACP	54.9	88.8	87.7	22.6	76.1
ACPred-FL	69.5	85.8	85.3	25.9	85.1
PEPred-Suite	68.3	90.6	89.9	32.0	86.1
ACPred-Fuse	72	89.5	89	32.0	86.8
AntiCP_ACC	68.3	88.5	87.9	28.8	85.3
AntiCP_DC	68.3	82.6	82.2	22.3	83.0
Hajisharifi’s	69.5	88.4	87.9	29.2	85.5
ACP-MCAM	**85.4**	**96.3**	**90.9**	**82.2**	**94.8**

Note: The best results are marked in bold and the second best results are underlined.

### Parameter Analysis

Several important parameters may affect the performance of our models, such as the learning rate and the kernel of multi-kernel CNN. Learning rate is an important parameter of deep learning. Through the adjustment of learning rate, we can see whether the objective function can quickly converge to the minimum value and fall into the local optimal value. An appropriate learning rate can make the objective function converge to the optimal value quickly.

In this section, we will perform a sensitivity analysis on these parameters. In our model, the number of training epochs is set to 50. The output dimension is 64. We train our model by modifying the learning rate. Table 4 shows that as the learning rate changes, the performance first gradually increases and then decreases. If the learning rate is equal to 2e-4, three of the five main evaluation indexes are the best. Accuracy, AUC, and F1-score are the highest. The model has achieved the best performance.

**TABLE 4:** The performance of the ACP-MCAM model affected by the learning rate.

Learning Rate	Accuracy	Precision	Recall	F1-Score	AUC
1e-4	0.8292	0.8	0.878	0.8372	0.9225
2e-4	0.9085	0.9589	0.8536	0.9032	0.9479
3e-4	0.8841	0.8888	0.878	0.8834	0.9341
4e-4	0.8902	0.9	0.878	0.8888	0.9388
5e-4	0.8841	0.8705	0.9024	0.8862	0.9375
6e-4	0.8902	0.8902	0.8902	0.8902	0.9298
7e-4	0.9085	0.9135	0.9024	0.9079	0.9301
8e-4	0.8902	0.9102	0.8658	0.8875	0.9144
9e-4	0.8841	0.8987	0.8658	0.8819	0.9207
1e-3	0.8658	0.8571	0.878	0.8674	0.9162

Note: The best results are highlighted in bold.

Since the CNN kernel represents several amino acids on an anti-cancer peptide sequence sharing the same parameters during the process of convolution. Therefore, the different combination of kernels means that the sequence of the anticancer peptide is affected by different combinations of several amino acids. Modifying the combination of the kernels may improve the effect of the model. Therefore, the combination of the kernel is also a very important parameter. As shown in Table 5, when the combination of kernels = (Li et al., 2006; Plumb et al., 2019; Song et al., 2020), the model achieved the best effect. Four of the five main evaluation indexes are the best. Accuracy, precision, AUC, and F1-score are the highest. This means that when using multi-kernel CNN to extract features from the model, selecting 1, 3, and 5 amino acid combinations for convolution calculation, and then combining these three features to obtain the best model effect. Our model adopts the combination of one, three, and five amino acids, which is better than considering all the amino acid sequences or just considering the properties of a single amino acid. This is the excellence of the CNN model.

**TABLE 5:** The performance of the ACP-MCAM model affected by kernel combination.

Kernel	Accuracy	Precision	Recall	F1-Score	AUC
1	0.8536	0.8372	0.878	0.8571	0.9177
3	0.8475	0.8275	0.878	0.852	0.932
5	0.8353	0.8021	0.8902	0.8439	0.9143
7	0.8475	0.8131	0.9024	0.8554	0.9158
1 + 3	0.8658	0.8488	0.8902	0.869	0.9439
1 + 5	0.8597	0.8831	0.8292	0.8553	0.9244
1 + 7	0.8109	0.8591	0.7439	0.7973	0.8856
3 + 5	0.8536	0.8536	0.8536	0.8536	0.9118
3 + 7	0.7987	0.7752	0.8414	0.807	0.9015
5 + 7	0.817	0.8095	0.8292	0.8192	0.9028
1 + 3+5	**0.9085**	**0.9589**	**0.8536**	**0.9032**	**0.9479**
1 + 3+7	0.8597	0.8831	0.8292	0.8553	0.9074
1 + 5+7	0.8353	0.8666	0.7926	0.828	0.9131
3 + 5+7	0.8719	0.8765	0.8658	0.8711	0.9434
1 + 3+5 + 7	0.8719	0.8961	0.8414	0.8679	0.9158

Note: The best results are highlighted in bold.

### Ablation Experiments

**FIGURE 3:**
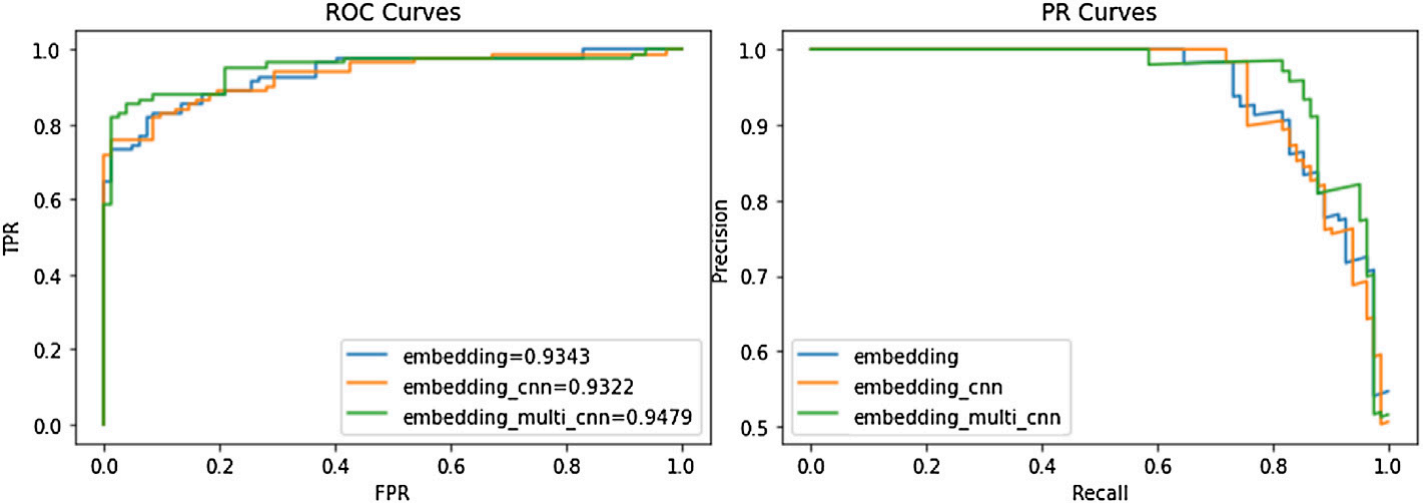
Performance comparison of ACP-MCAM and existing methods. The left figure is the ROC curves of different models on the ACP dataset. The right figure is the PR curve of different models on the ACP dataset.

We compared our model to the ACP164 data set for ablation experiments. It can be seen that the experiment is mainly to compare three models: the embedding attention model, CNN attention model, and Multi-kernel CNN attention model. From Table 6 and Figure 3, we can observe the performance comparison of three different embedding methods. In general, the performance of multi-kernel CNN is better than that of all existing methods on the ACP dataset, indicating that Multi-kernel CNN’s embedding method is more powerful than models based on other embedding features.

**TABLE 6:** The performance of three different models on three different peptide datasets.

Model	Accuracy	Precision	Recall	F1	AUC
Embedding + ACP	0.8719	0.9178	0.817	0.8645	0.8719
Embedding_cnn + ACP	0.8658	0.8947	0.8292	0.8607	0.9321
Embedding_multicnn + ACP	0.9085	0.9589	0.8536	0.9032	0.9479
Embedding + NPs	0.8343	0.8197	0.8571	0.8380	0.8840
Embedding_cnn + NPs	0.8229	0.8192	0.8286	0.8239	0.8894
Embedding_multicnn + NPs	0.8400	0.8479	0.8285	0.8381	0.9063
Embedding + AFPs	0.8625	0.8371	0.9003	0.8675	0.9161
Embedding_cnn + AFPs	0.9038	0.8803	0.9347	0.9067	0.9580
Embedding_multicnn + AFPs	0.8762	0.8119	0.9793	0.8878	0.9677

Note: The best results of different dataset are highlighted in bold.

Figure 3 is a more intuitive comparison between the several embedding methods of our model, including the ROC curve and precision-recall (PR) curve. From Figure 3, we can see that in both the ROC curve and PR curve, the embedding method of the embedding_multi_cnn can achieve the best effect of the model. Note that the experimental results in this section only reflect the performance of the model on the ACP data set, and it is difficult to avoid certain deviations. Therefore, we evaluate the performance of this model through experiments on other peptide datasets (NPs and AFP). The dataset is shown in Table 1. NPs1400 was used as the training set, NPs350 was used as the test set; AFPs2336 was used as the training set, and AFPs582 was used as the test set to verify the model.

The results in Table 6 show that Multi-kernel CNN performs best on the ACP dataset and AFPs dataset, especially in ACC and AUC. On the NPs data set, CNN has the best feature extraction effect. Therefore, it can be inferred that on the NPs data set, every three consecutive amino acids were regarded as an amino acid group for classification prediction to achieve the best effect.

### Feature Representations and Visualization

**FIGURE 4:**
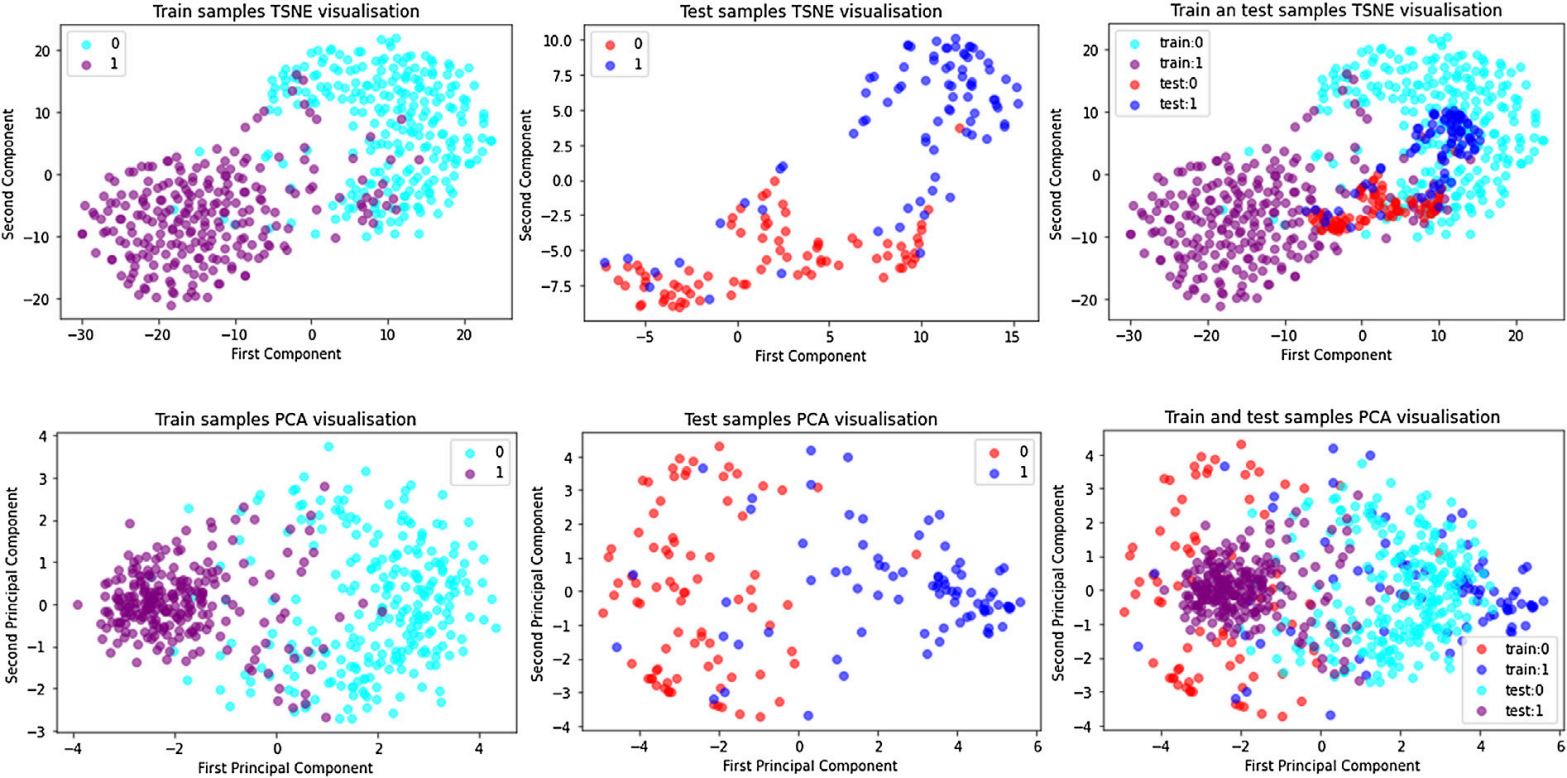
Dimension reduction of each samples on ACP500 and ACP164 dataset by TSNE and PCA.

Principal Component Analysis (PCA) (Smith, 2002) is a common linear dimensionality reduction method, while t-distributed Stochastic Neighbor Embedding (TSNE) (Laurens and Hinton, 2008) is a non-linear dimensionality reduction method. Due to different principles and mechanisms, TSNE runs slower, while PCA is relatively fast. PCA transforms a set of potentially correlated variables into a set of linearly uncorrelated variables through orthogonal transformation, and the transformed set of variables is called principal components. The idea of PCA is to map n-dimensional features to k-dimensions (k < n), which are brand new orthogonal features. In this paper, k is equal to 2. The basic idea of TSNE is that similar data points in high-dimensional space map to similar distances in low-dimensional space. The attribute information retained by TSNE is more representative and can relatively reflect the differences between samples. To visually verify the effectiveness of the ACP-MCAM model and improve the interpretability of the model, this paper uses principal component analysis (PCA) and t-distributed stochastic neighborhood embedding (TSNE) to learn the high-dimensionality of ACP sequences at different stages. The high-dimensional feature representation vectors of anticancer peptide sequences at different stages are reduced to a two-dimensional plane for easy visualization, and the results are shown in Figure 4.

The figure shows that in the ACP dataset, the positive examples (represented by train:1 and purple dots) and negative examples (represented by train:0 and blue dots) in the training dataset are mixed in the initial stage because they are initialized randomly. The same is in the test dataset (positive examples are represented by test:0 and red dots, and negative examples are represented by test:1 and blue dots), which indicates that the model has no distinguishing ability before training. As the training epoch number increases, positive and negative samples are gradually separated from the sample points. We can observe that in the training dataset and the test dataset, the embedding vectors of the ACP samples almost belong to the same cluster, and after training, the positive and negative examples have similar distributions, which indicates that the model has indeed learned feature of the positive and negative samples. This shows that the model in this paper can learn the common features and distinguishing features of positive and negative cases.

In addition, there are many ACPs in the negative cluster, but few non-ACPs in the positive cluster, which explains the reason why the performance of SP is better than SE to some extent. We speculate that those ACPs predicted to be negative samples have characteristics that our method cannot capture. Therefore, the unique physical and chemical properties of these indistinguishable samples should be further studied in the future.

## Conclusion

A very important point in deep learning is how to extract features from data. The quality of the extracted features will largely affect the effectiveness of the model. The advantage of natural language processing (NLP) is that it can effectively extract word embedding and sequence information from sequence data, and use it for subsequent specific tasks. Our method can automatically learn useful information from the amino acid sequence data of anti-cancer peptides and perform feature representation on node features and sequence features.

In this work, we proposed a new predictive model called ACP-MCAM. This is a powerful bioinformatics tool. The model can predict anti-cancer peptides based on a convolutional neural network and self-attention mechanism network, which can extract effective amino acid nodes and anti-cancer peptide sequence information. The advantage of ACP-MCAM is that it can effectively use the position information and the information of the amino acid node cluster. The ACP-MCAM model mainly includes the following modules: embedding layer, multi-kernel convolutional neural network layer, position coding layer, attention encoding layer, and task output layer. The experimental results of 10-fold cross-validation and independent testing show that this predictor can effectively distinguish anti-cancer peptides from non-anti-cancer peptides. Moreover, we used the model to predict neuropeptides and antifungal peptides and achieved good prediction results. The excellent predictive ability of this model will accelerate its application in cancer treatment.

Our model has achieved good prediction performance, but there are still some shortcomings to be overcome. First of all, the prediction performance of the model fluctuates greatly, and different parameters have a greater impact on the prediction results. The main reason is that 500 pieces of data in the training set and 164 pieces of data in the test set are too small for deep learning to train all parameters. This is where we will strive to improve in the future. In future work, we will expand more datasets and try more computing techniques, such as pre-training strategies for automatic feature extraction, to achieve more accurate and better predictions. Second, in our current case study, we only made computer model predictions based on the original database and did not verify it in silicon experiments. In the future, we will plan to cooperate with biologists to conduct wet laboratory experiments to verify the predicted results.

## Data Availability

The original contributions presented in the study are included in the article/Supplementary Material, further inquiries can be directed to the corresponding authors.
